# Accelerating effect of Shilajit on osteogenic property of adipose-derived mesenchymal stem cells (ASCs)

**DOI:** 10.1186/s13018-022-03305-z

**Published:** 2022-09-24

**Authors:** Parisa Kangari, Leila Roshangar, Aida Iraji, Tahereh Talaei-Khozani, Mahboobeh Razmkhah

**Affiliations:** 1 Student Research Committee, Shiraz University of Medical Sciences, Shiraz, Iran; 2 Department of Tissue Engineering and Applied Cell Sciences, School of Advanced Medical Sciences and Technologies, Shiraz University of Medical Sciences, Shiraz, Iran; 3 Shiraz Institute for Cancer Research, School of Medicine, Shiraz University of Medical Sciences, Shiraz, Iran; 4 Stem Cell Research Center, Tabriz University of Medical Sciences, Tabriz, Iran; 5 Central Research Laboratory, Shiraz University of Medical Sciences, Shiraz, Iran; 6 Stem Cell and Transgenic Technology Research Center, Shiraz University of Medical Sciences, Shiraz, Iran; 7 Histomorphometry and Stereology Research Center, Shiraz University of Medical Sciences, Shiraz, Iran; 8 Tissue Engineering Laboratory, Department of Anatomy, School of Medicine, Shiraz University of Medical Sciences, Shiraz, Iran

**Keywords:** Osteogenic differentiation, Shilajit, Alginate, Adipose-derived mesenchymal stem cells, Regenerative medicine

## Abstract

**Background:**

Shilajit has been widely used remedy for treating a numerous of illness such as bone defects in Iran traditional folk medicine since hundreds of years ago. The aim of the present study was to explore the effect of Shilajit on the osteogenic differentiation of human adipose-derived mesenchymal stem cells (ASCs) in two- (2D) and three-dimensional (3D) cultures.

**Materials and methods:**

ASCs were seeded in 3D 1% alginate (Alg) hydrogel with or without Shilajit (500 µg/mL) and compared with 2D cultures. Then, characterization was done using electron microscopy (SEM)/energy-dispersive X-ray spectroscopy (EDX), alkaline phosphatase (ALP) activity, alizarin red staining and Raman confocal microscopy.

**Results:**

Adding Shilajit had no impact on the Alg scaffold degradability. In the 3D hydrogel and in the presence of osteogenic medium (OM), Shilajit acted as enhancer to increase ALP activity and also showed osteoinductive property in the absence of OM compared to the 2D matched groups at all time points (days 7 and 21 both *P* = 0.0006, for 14 days *P* = 0.0006 and *P* = 0.002, respectively). In addition, calcium deposition was significantly increased in the cultures exposed to Shilajit compared to 2D matched groups on days 14 (*P* < 0.0001) and 21 (*P* = 0.0003 and *P* = 0.003, respectively). In both 3D and 2D conditions, Shilajit induced osteogenic differentiation, but Shilajit/Alg combination starts osteogenic differentiation in a short period of time.

**Conclusion:**

As Shilajit accelerates the differentiation of ASCs into the osteoblasts, without changing the physical properties of the Alg hydrogel, this combination may pave the way for more promising remedies considering bone defects.

## Introduction

Bone defects are one of the most common mobility disabilities that cause notable health, social and economic problems. Annually, more than 20 million individuals are at risk for bone loss [1]. In the past decades, bone substitutes such as allografts, xenografts and alloplastic materials for bone defect repair have been presented; however, they may have serious limitations and side effects. In recent years, to overcome the impediments and disadvantages associated with bone grafts, regenerative medicine approaches including cell therapy have been extensively developed [2]. Mesenchymal stem cells (MSCs) are a convenient option for cell therapy in different diseases such as bone defects. In this regard, extensive investigations have been performed on MSC therapy in bone repair alone or in combination with other remedies [3, 4]. Interestingly, there are reports showing that adipose-derived mesenchymal stem cells (ASCs) are an appropriate source for the treatment of skeletal disorders such as musculoskeletal diseases and shoulder pains [5–7]. Systemic and local administration of allogeneic bone marrow-derived mesenchymal stem cells (BM-MSCs) in rat femoral bone fracture led to an elevation in the callus formation and osteoblast differentiation [8]. In another study, a combination of amniotic fluid-derived MSC and platelet-rich plasma (PRP) embedded in polycaprolactone scaffolds significantly improved rat cranial critical size defect, collagen type I expression and neoangiogenesis [9]. The positive impacts of MSCs on bone regeneration can be reinforced by some natural or synthetic products. For instance, in vitro studies demonstrated that curcumin [10], thymoquinone [11], polydatin [12], acemannan [13], and catalpol [14]can boost osteogenic potential of MSCs.

Shilajit, also named mineral pitch, is a herbomineral natural substance that is produced from deposition plant materials such as Euphorbia and Trifolium plants and lichen and is one of the most widely applicable compositions with numerous therapeutic efficacies in traditional folk medicine. Shilajit is widely distributed in some parts of the world including Iran, Altai Mountains [15] and Australia [16] with mainly same chemical compositions. It contains organic compounds (60–80%), inorganic ingredients (20–40%) and various elements [17]. Based on previous studies, the main chemical compounds of Shilajit are fulvic acid, dibenzo-alpha-pyroons (DBPs) and DBP chromoproteins. Additionally, the overall mineral content of Shilajit is mainly composed from potassium, calcium and magnesium, while sulfur and sodium being the next [18]. Also, Shilajit contains exogenous amino acids, such as methionine, leucine and threonine, and also endogenous amino acids such as histidine, proline, glycine, tyrosine, arginine and aspartic acid [17]. It has been revealed that Shilajit has various biological activities such as immunomodulatory, anti-inflammatory, antimicrobial and antioxidant. It also has gastroprotective, anticancer, spermatogenesis, oogenesis and anti-hyperlipidemia effects [19, 20].The Shilajit antioxidant activity can be due to the presence of DBPs and fulvic acid. Besides, fulvic acid facilitates importing minerals into the target cells, protecting the electrical potential and preventing cell death [21, 22]. The safety and healing efficacy of Shilajit has been studied in humans and animals [18].The Shilajit compounds exhibit a great potential for healing the different diseases such as bone fractures, osteoarthritis [23, 24], anemia [25], diabetes [26] and Alzheimer [27]. A clinical trial conducted in Iran revealed that oral administration of Shilajit after tibia fracture surgery accelerated bone repair [28]. The findings of another cohort study in Iran indicated that Shilajit consumption had beneficial impact on femur and tibia bone fracture repair [29]. In addition, according to the results of a randomized double-blinded study, Shilajit was an effective treatment for ameliorating pain in osteoarthritis dog model due to its anti-inflammatory effects [24]. Another study showed that Shilajit is an effective treatment for chronic ulcers [30].

Although the positive effects of systematic and local administration of Shilajit on bone fracture repair was documented in traditional medicine, there are a few studies showing the effect of Shilajit on osteogenic capacity of MSCs. In the current study, the osteogenic potential of human adipose-derived MSCs (ASCs) was compared in the presence or absence of Shilajit in both 2D and 3D culture systems.

## Materials and methods

### Preparation of Shilajit

Shilajit was collected from Lorestan province altitudes, in western of Iran. Fifty grams powdered Shilajit was dissolved in 500 mL distilled water. This mixture was boiled on high flame to melt, and then the flame was declined until it becomes concentrated and black. Shilajit was stored at room temperature and protected from moisturizing. To prepare the appropriated concentrations, the Shilajit extract was dissolved in media or alginate (Alg) and then was sterilized through 0.22-µm syringe filter (Jet Biofil, China).

### Inductively coupled plasma mass spectrometry (ICP-MS)

The mineral contents of Shilajit were analyzed by inductively coupled plasma mass spectrometry (ICP-MS) [31]. First the sample was crushed into fine powder, and then 0.1 gr was treated with concentrated HNO_3_ and allowed to stand for 30 min in room temperature. Five hundred liters of concentrated HClO_4_ was added and heated at 100 °C. During the heating process, 200–300 µL of concentrated hydrofluoric acid (HF) was added gradually until a clear solution was obtained. This solution was finally used for analysis via ICP spectrometer (ELAN 9000, Perkin-Elmer SCIEX).

### Determination of antioxidant capacity

The 1,1-diphenyl-2-picrylhydrazyl (DPPH) method is based on the spectrophotometric measurement of DPPH° concentration at maximum absorption wavelength at 517 nm. The antioxidant molecules can quench DPPH free radicals (reduce the absorption) and convert them to a colorless or bleached product [32].

The DPPH radical-scavenging activity of Shilajit was determined according to the method of Brand-Williams with some modifications [33]. Fifty microliters of various concentrations of Shilajit )10, 5,2.5,1.25, 0.625 mg/mL) (dissolved in deionized water) was added to 100 μl methanol containing 0.05 mg/mL of DPPH radical in 96 well plates. The samples were incubated for 20 min at room temperature in dark, and then absorbance was measured with spectrophotometer at 517 nm wavelength.

The experiment was carried out triplicate. Radical-scavenging activity was calculated using the following formula:

$$\% {\text{Inhibition}} = \left[ {\left( {{\text{Ab}} - {\text{Ae}}} \right)/{\text{Ab}}} \right] \, \times {1}00$$

where Ab is the absorbance of the blank sample and Ae is the absorbance of Shilajit.

### Proton nuclear magnetic resonance (H-NMR)

Shilajit sample was vortexed for about 5 min in D_2_O at room temperature to completely dissolve, and the sample was then filtered to remove any particle. The H-NMR spectra of Shilajit were recorded on a Bruker Ascend 300 spectrometer (Bruker BioSpin GmbH, Rheinstetten, Germany) equipped with a 5-mm probe. Briefly, the spectra were referenced relative to the solvent residual peak (4.7 ppm) with 1024 number of scans, 2 number of dummy scans, 1-s relaxation delay, 24,670 time domain, 2.05 acquisition time, and the other parameters were set as default. Assignments of different protons were done based on the chemical shift corresponding to solvent peak (lock frequency) as well as the integrated intensity (proportional to the number of hydrogen) and coupling constant (spin–spin splitting) [34].

### Cell isolation and culture

Adipose tissues were collected from the two healthy female patients aged 30 years underwent liposuction surgery and transferred to Cancer and Stem Cell Laboratory of Shiraz Institute for Cancer Research (ICR), Shiraz University of Medical Sciences. The tissues were washed with sterile 1 × phosphate buffer saline (PBS) containing 1% of penicillin/streptomycin (P/S, Bioidea, Iran) to remove contaminating blood cells. Then they were cut into small pieces and incubated in 0.2% collagenase type I (Gibco, USA) for 30 min at 37 °C and 5% CO_2_. After digestion, an equal volume of Dulbecco's modified eagle medium (DMEM, Bioidea, Iran) supplemented with 10% heat inactivated fetal bovine serum (FBS, Gibco, USA) and 1% P/S was added to the samples to neutralize the collagenase type I activity.

Then the obtained soup was centrifuged, and cell pellet containing ASCs were resuspended in DMEM supplemented with 20% FBS and 1% P/S. The medium was changed every 3 days, and the cells were passaged using 0.25% trypsin solution (Bioidea, Iran). ASCs from passage 3 were used for subsequent experiments.

### Characterization of ASCs: morphology and phenotype

Cell morphology was demonstrated in cell cultures from passage 3 by crystal violet staining (Acros Organics, USA). Cells were stained with 0.5% crystal violet in methanol for 10 min at room temperature. Excess stain was rinsed with distilled water, and spindle-shaped cells were observed with the microscope.

Flow cytometry was performed to analyze the surface antigen expression of isolated ASCs. Briefly, ASCs were stained with fluorescein-5-isothiocyanate (FITC)-conjugated CD45, phycoerythrin (PE)-conjugated CD34 and CD105 and peridinin–chlorophyll–protein (PerCP)-conjugated CD73 antibodies (BD, Biosciences, USA) and were incubated at 4 °C for 15 min in the dark. Finally, the frequency of the positive cells for each CD marker was determined using FACS Calibur flow cytometry system (BD, Biosciences, USA). Isotype antibodies were used to exclude non-specific staining of the cells, and the data were analyzed by flowjo 7.6 software package (Ashland, San Diego CA, USA).

### MTT assay

3-(4,5 Dimethyl-2-thiazolyl)-2,5-diphenyl tetrazolium bromide) (MTT, Merck, Germany) was used to determine the appropriate and nontoxic dose of Shilajit. In short, 1 × 10^4^ ASCs/well in 100µL DMEM containing 10% of FBS and 1% of P/S on 96-well plates were incubated with a range of concentrations of Shilajit from 5000 to 9.7 µg/ml. After 24, 48 and 72 h, the medium was discarded, and 150 μl of 0.1% MTT solution in culture medium was added and incubated at 37 °C for 4 h. Afterward, MTT was removed, and 150 μL of dimethyl sulfoxide (DMSO, Sigma-Aldrich, USA) was added and incubated at room temperature for 1 h to dissolve the formazan crystal. Finally, the optical density (OD) of the formazan solution was measured with spectrophotometer at 492 nm wavelength.

### Scaffold preparation

Alginate (Alg) solution was prepared from low viscosity Alg (Sigma-Aldrich, USA) dissolved in PBS 1 × and stirred in a glass beaker overnight at room temperature (25 °C) at a final concentration of 1% w/v. For the cell assays, obtained solution was sterile filtered using 0.22-μm syringe filter and stored at 4 °C for further usage.

### Differentiation of ASCs

The osteogenic differentiation potential of ASCs was compared in different groups in the presence or absence of Shilajit in 2D and 3D cell culture.

For 3D cultures, 3 × 10^5^ ASCs/mL were suspended in 1% (w/v) Alg with or without 500 µg/ml Shilajit, and gelation was induced by adding 2.5% CaCl_2_. The cells were cultured with or without osteogenic medium (OM, Kiazist, Iran) supplementation for 7, 14 and 21 days. The 3D cultures were divided into four groups: (1) Shilajit/Alg/ASCs + OM, (2) Alg/ASCs + OM (positive control), (3) Shilajit/Alg/ASCs and (4) Alg/ASCs (negative control). The cells in negative control cultures received basic medium (DMEM containing 10% FBS and 1% P/S).

For 2D cultures, 3 × 10^4^ cells/mL were seeded in basic medium. Upon 70% confluency, a matched 2D cultures also was performed for 7, 14 and 21 days. The 2D treatments include with the cultures exposed to either OM and 500 µg/ml Shilajit at a ratio of 1/2, the same concentration of Shilajit in DMEM, positive control that received OM, or negative control. The media were changed every 3 days for both 2D and 3D groups.

### Mineralization assay

After 21 days of differentiation, morphological characterization of encapsulated ASCs within the hydrogels as well as calcium and phosphorus content was, respectively, evaluated using scanning electron microscopy (SEM) and energy-dispersive X-ray spectroscopy (EDX). Briefly, hydrogels were lyophilized, samples were fractured into pieces, and then, the cutoff was coated by gold replica using Q150R-ES sputter coater (Quorum Technologies, London, UK) and SEM imaging was taken using a VEGA3 microscope (TESCAN, Brno, Czech Republic) at 10 kV accelerating voltage. EDX was also performed to evaluate the amount of calcium and phosphorous within the scaffolds. For EDX, three repeats from every group were made.

To assess alkaline phosphatase (ALP) activity, total protein of the differentiated cells was extracted at 7, 14 and 21 days, and ALP colorimetric assay kit (Pars Aazmoon, Iran) was used to evaluate the ALP activity in lysates [35]. To do this, at each point of time, the culture medium was aspirated and the cells were washed with PBS. For extraction of total protein, cells were lysed in the buffer containing 0.2% Triton X-100 in 20 mM Tris–HCl on ice for 15 min. For 3D cultures, the Alg scaffolds were first exposed to 55 mM sodium citrate for depolymerization at 25 °C for 25 min. Then, the cells were lysed by adding the same lyses buffer. The cell lysate was centrifuged at 2500 g at 4 °C for 15 min, and the supernatant was used to assay ALP activity. Total protein was finally used for normalizing the enzyme activity level (U).

Alizarin red S staining was applied to evaluate the calcium deposition. After 7, 14 and 21 days of differentiation, culture medium was aspirated, and the 2D and 3D cultures were washed in PBS and fixed in 10% formaldehyde for 30 min. Following double washes with PBS, the samples were stained with 2% alizarin red S (Sigma-Aldrich) for 10 min. Additionally, quantitative analysis of calcium content was performed by adding 500 μL of 100 mM cetylpyridinium chloride monohydrate (Merck, Germany) to elute alizarin red S. Then absorbance was evaluated at 405 nm [11].

### Confocal Raman microscope

After 21 days of differentiation, all 3D samples were lyophilized and fractured into pieces, and then, the Raman spectra of the cell laden scaffolds along with Shilajit were obtained by Lab-Ram HR Confocal Raman spectrometer (Horiba, Japan). Both Shilajit and 3D samples were excited with laser power level at 50 mW using the excitation laser with the wavelength of 785 nm with 100 mV power for Raman spectra recording. We analyzed the Raman shift at the range of 500–2000 cm^−1^ [36].

### Scratch assay (wound healing assay)

ASCs were seeded at 3 × 10^4^ cells/well and permitted to adhere and form a confluent. A scratch as a straight line through the center of each well was formed by a sterile 10-μl pipette tip, and the wells were washed with PBS to remove cell debris. After that, 500 μL of DMEM with or without Shilajit was added to each well as treated and untreated groups. The staining was done with crystal violet at zero, 8 and 12 h after incubation. The cell migration to the scratch was evaluated in both treated and untreated groups using the Image j software (https://imagej.nih.gov/ij/index.html). The cell-free area in each group was calculated at each point of time [37].

### Biodegradation test

The in vitro biodegradation test was carried out to evaluate the possible impact of Shilajit on the degradation rate of Alg hydrogel scaffolds. To do this, 1% Alg hydrogels were prepared in the present or absence of Shilajit and electrogellate by 2.5% CaCl_2_ (*n* = 3). Prior to enzyme application, the scaffolds were weighed. Then 500 µL of 0.01% trypsin (Bioidea, Iran) was added and incubated at 37 °C for 12, 24, 48, 72, 100 and 122 h. At the selected time points, trypsin was completely removed and experimental plates weighed. The enzymatic degradation rate was calculated by subtracting the start and end weight of the scaffolds.

### Statistical analysis

Graph Pad Prism software version 8.1 was used to analysis and depicted the graphs. One-way ANOVA and two-way ANOVA were applied for compare the data from multiple groups. The results were displayed as mean ± standard error mean (SEM), and *p* value < 0.05 was defined as significant. All experiments were performed in triplicate.

## Results

### Inorganic composition of Shilajit

ICP-MS showed the presence of the high level of calcium (Ca), aluminum (Al), potassium (k), magnesium (Mg) and sodium (Na) (all 100 ppm). A lower amount of the other elements such as sulfur (S), phosphorus (P) and manganese (Mn) (50, 10 and 5 ppm, respectively) was found in Shilajit. Table 1 indicates that the amount of the other minerals was the least.

**Table 1:** Elemental composition of Shilajit

Elements	Concentration of elements (ppm)
Al	100
Ca	100
Fe	100
K	100
Na	100
S	50
P	10
Ti	10
Zr	5
Mn	5
Ba	1
Co	1
Cr	1
Cu	1
Li	1
Zn	1
La	1

### Antioxidant activity

The antioxidant activity of Shilajit was calculated as the percentage-scavenging activity against DPPH radical. Shilajit showed 49.84 ± 1.96% and 30.07 ± 0.24% of DPPH inhibition at 5 and 2.5 mg/mL, respectively. The results indicated that Shilajit inhibited free radicals at the tested concentrations.

### H-NMR spectrum

**Fig. 1:**
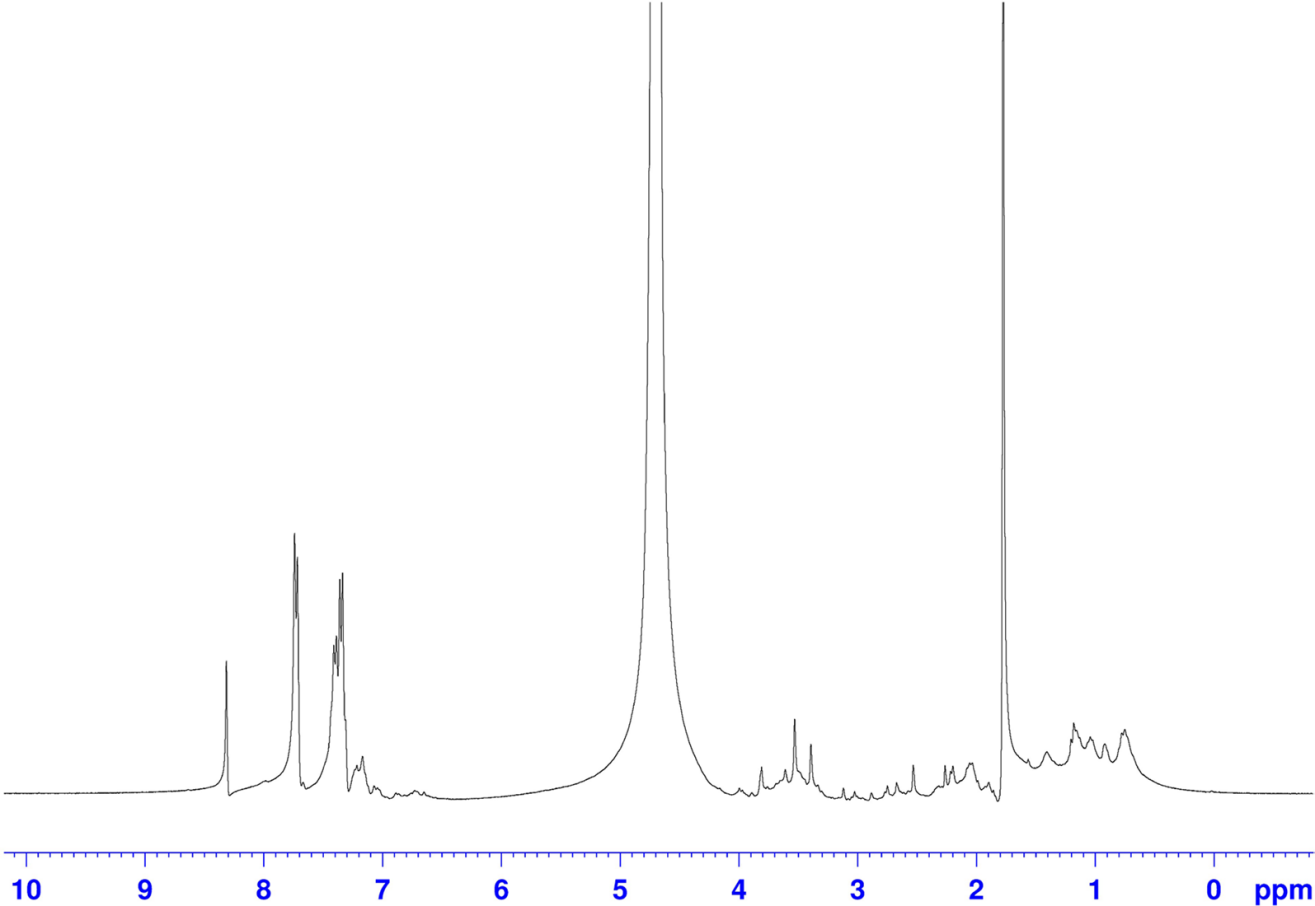
H-NMR spectrum of Shilajit. The H-NMR spectra shows the presence of functional groups related to organic compounds, especially polysaccharides in Shilajit

The H-NMR spectra of the Shilajit are depicted in Fig. 1. To better analyze the H-NMR results, the spectra are divided into four main regions. The first region, from 0 to 1.8 ppm, is generally assigned to protons on methyl and methylene carbons directly bonded to other carbons. The second region, from 1.8 to 3.0 ppm, can be assigned to protons on methyl, methylene and methane carbons which are bonded to electronegative functional groups such as aromatic rings or carbonyls moieties. The third one is chemical shift from 3.0 to 4.5 that belongs to the polysaccharide. The fourth region (6.0–8.0 ppm) is assigned to the presence of both aromatic and heteroaromatic protons with the contribution of the unsaturated groups [34, 38, 39].

### Phenotype and morphological identification of ASCs

**Fig. 2:**
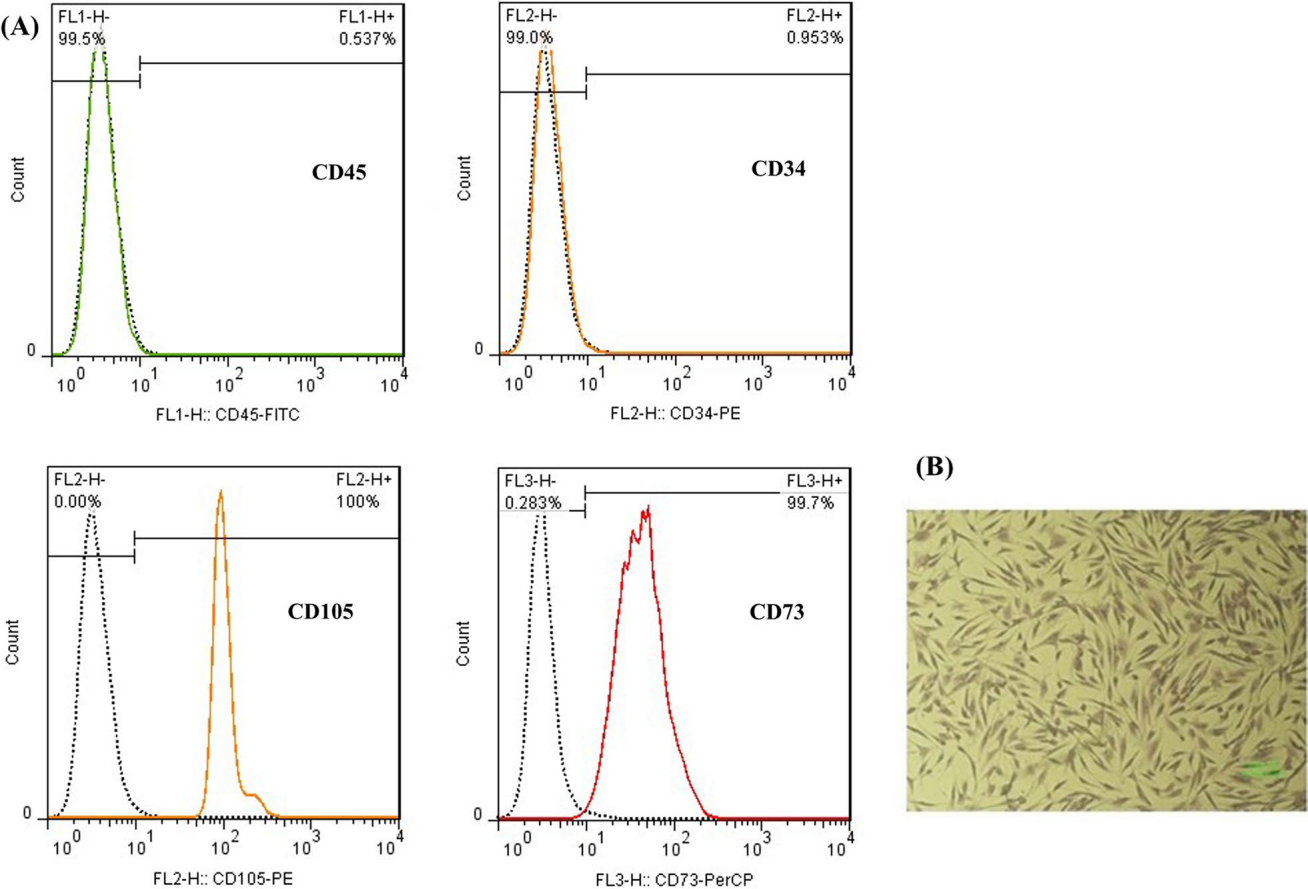
MSC characterization. **A** Flow cytometry indicated that ASCs were negative for CD45 and CD34 but positive for CD105 and CD73. **B** Isolated ASCs showed fibroblast-like shapes

Phenotypic characterization of cultured ASCs at passage 3 was assessed using flow cytometry. The results demonstrated that the cells expressed mesenchymal markers such as CD73 and CD105, while they were negative for hematopoietic markers such as CD34 and CD45 (Fig. 2A). Additionally, the crystal violet staining of ASCs was performed and microscopic observations with light inverted microscope validated their spindle and fibroblast-like morphology (Fig. 2B).

### Cytotoxicity assessment

To obtain the appropriate dose of Shilajit, the survival rate of ASCs in the presence of different concentrations (9.7–5000 μg/mL) of Shilajit was evaluated. As MTT test showed supplementation of 500 μg/mL of Shilajit had no cytotoxic effect, and so, this concentration was used for in vitro investigations.

### Mineralization assessments

**Fig. 3:**
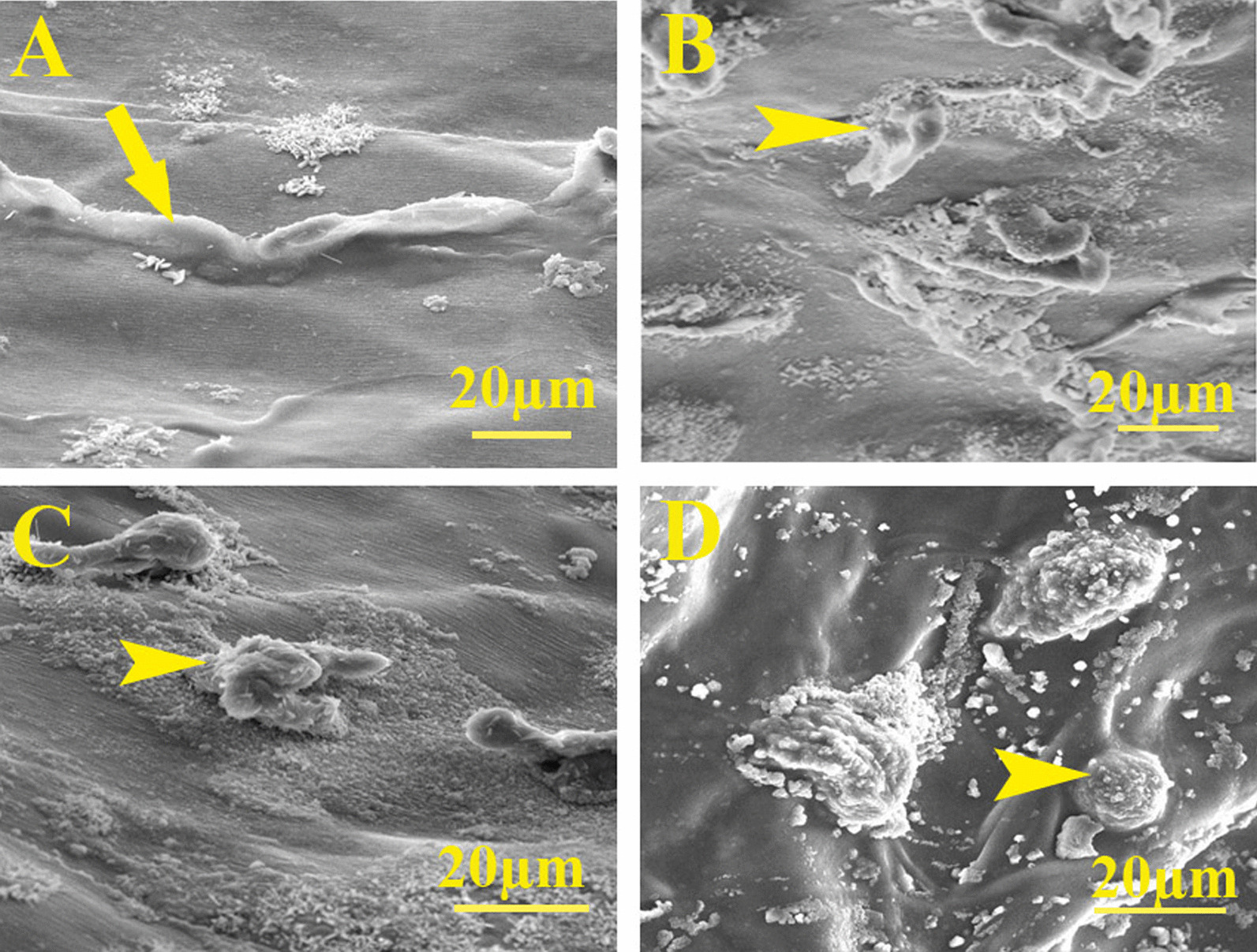
Scanning electron micrographs. **A** Cells with mesenchymal stem cell phenotype expanded in Alg/ASCs. Osteoblast-like cells with short processes on **B** Shilajit/Alg/ASCs + OM, **C** Shilajit/Alg/ASCs and **D** Alg/ASCs + OM conditions

SEM images revealed the round, osteoblast-like cells with few filopodia for the cells encapsulated in Shilajit/Alg with or without OM and Alg/ASCs + OM. However, in the absence of both Shilajit and OM, the cells were spindle and fibroblast-like shape, the typical phenotype of MSCs (Fig. 3).

**Fig. 4:**
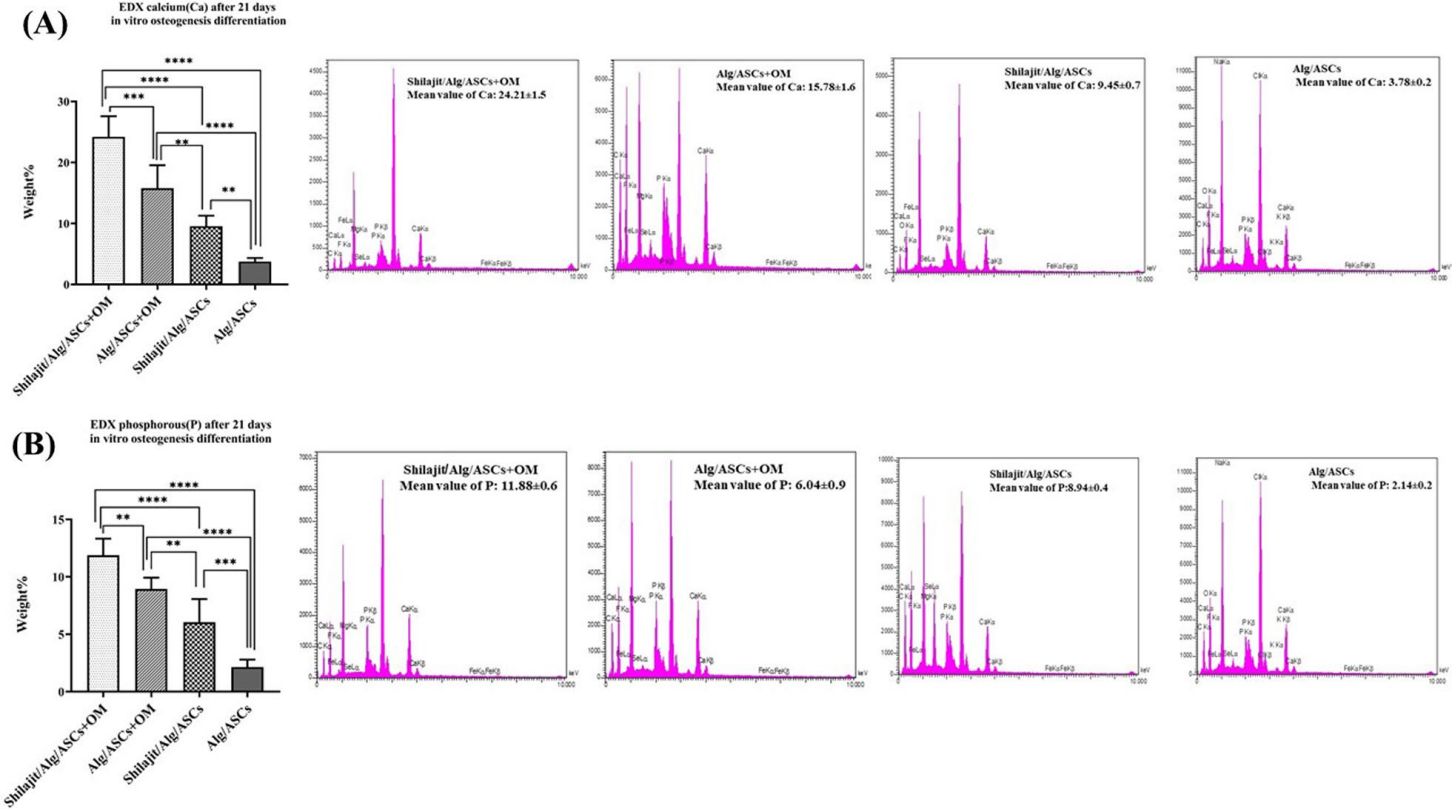
Energy-dispersive X-ray spectroscopy analysis. Calcium (Ca, **A**) and phosphorus (P, **B**) contents in 3D groups after 21 days. The highest level of Ca and P was observed on Shilajit/Alg/ASCs + OM. The strongest peaks of Ca and P were shown on Shilajit/Alg/ASCs + OM (**P < 0.01, ***P < 0.001, ****P < 0.0001)

According to the results from EDX analyses, the highest level of calcium and phosphorus deposition was observed in the Shilajit/Alg/ASCs + OM (*P* = 0.0001 and *P* = 0.004 for Alg/ASCs + OM, respectively), (both *P* < 0.0001 for Shilajit/Alg/ASCs and Alg/ASCs). In addition, Alg/ASCs + OM presented a significant increase in the level of calcium and phosphorous deposition compared to both Shilajit/Alg/ASCs (*P* = 0.002 and *P* = 0.004; respectively) and Alg/ASCs (for both *P* < 0.0001). However, the amount of calcium and phosphorous was higher in the Shilajit/Alg/ASCs than Alg/ASCs (*P* = 0.003 and *P* = 0.0004; respectively) (Fig. 4A, B).

**Fig. 5:**
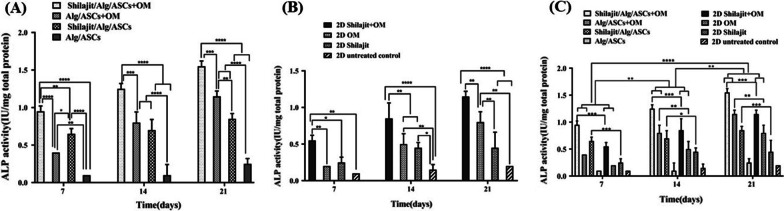
The ALP activity assessment. The cells were cultured in 3D (**A**) and 2D (**B**) osteogenic conditions on 7, 14 and 21 days after differentiation and also, comparing 3D and 2D cultures was done on these time points (**C**). The highest level of ALP activity was indicated in Shilajit/Alg/ASCs + OM on 21 days after differentiation compared with other groups (*P < 0.05, **P < 0.01, ***P < 0.001, ****P < 0.0001)

The ALP activity was estimated in the various experimental groups on days 7, 14 and 21. ALP activity was significantly higher in the cultures received both Shilajit and OM on 7 (*P* < 0.0001), 14 )*P* = 0.0003) and 21 (*P* = 0.0007) days compared with Alg/ASCs + OM that indicates the boosting impact of Shilajit on OM. Although the Shilajit had osteoinductive property in all points of time, the Shilajit was not as effective as OM in elevating ALP activity. Therefore, ALP activity displayed a significantly elevation in Shilajit/Alg/ASCs + OM compared with Shilajit/Alg/ASCs (*P* = 0.005 for 7 days; *P* < 0.0001 for 14 and 21 days). In addition, Shilajit/Alg/ASCs could significantly elevate the ALP activity compared to Alg/ASCs without OM (*P* < 0.0001 for all time points) or with OM on day 7 (*P* = 0.015), while at day 21 ALP activity increased in Alg/ASCs + OM than Shilajit/Alg/ASCs (*P* = 0.005). Indeed, ALP activity increased in Alg/ASCs + OM compared with Alg/ASCs (*P* = 0.005 for day 7; *P* < 0.0001 for days 14 and 21) (Fig. 5A).

In 2D conditions, Shilajit supplementation also boosted the osteogenic effect of OM as ALP activity increased significantly in Shilajit + OM treated compared to the cultures treated with either OM (*P* = 0.009, all time points) or Shilajit alone (for day 7 *P* = 0.021, day 14 *P* = 0.004 and day 21 *P* < 0.0001) and untreated control (for 7days P = 0.0019, for 14 and 21 days P < 0.0001). On days 14 and 21, Shilajit also caused a significant increase in ALP activity even in the absence of OM compared to the negative control cultures (P = 0.004 and *P* = 0.021, respectively; Fig. 5B).

Comparing 3D and 2D cultures displayed that ALP activity was significantly higher in 3D culture conditions than matched groups of 2D conditions except for negative matched groups. The ALP activity of the cells encapsulated in Shilajit/Alg/ASCs + OM, and Shilajit/Alg/ASCs was higher compared to the 2D matched groups at all time points (days 7 and 21 both *P* = 0.0006, for 14 days P = 0.0006 and *P* = 0.02, respectively). In addition, Alg/ASCs + OM for 14 and 21 days exhibited significant higher ALP activity than 2D condition (*P* = 0.007 and *P* = 0.002, respectively). As the time progress, the 3D and 2D cultures in presence both Shilajit and OM showed a significant enhancement in ALP activity on day 14 compared to the same groups on days 7 (P = 0.007 for Shilajit/Alg/ASCs + OM, P = 0.0006 for Alg/ASCs and P = 0.007 for both 2D Shilajit + OM and negative control groups) and 21 as well as the same groups on days 7(P < 0.0001) and 14(P = 0.007 for Shilajit/Alg/ASCs + OM, P = 0.002 for Alg/ASCs and *P* = 0.007 for both 2D Shilajit + OM and negative control groups; Fig. 5C).

**Fig. 6:**
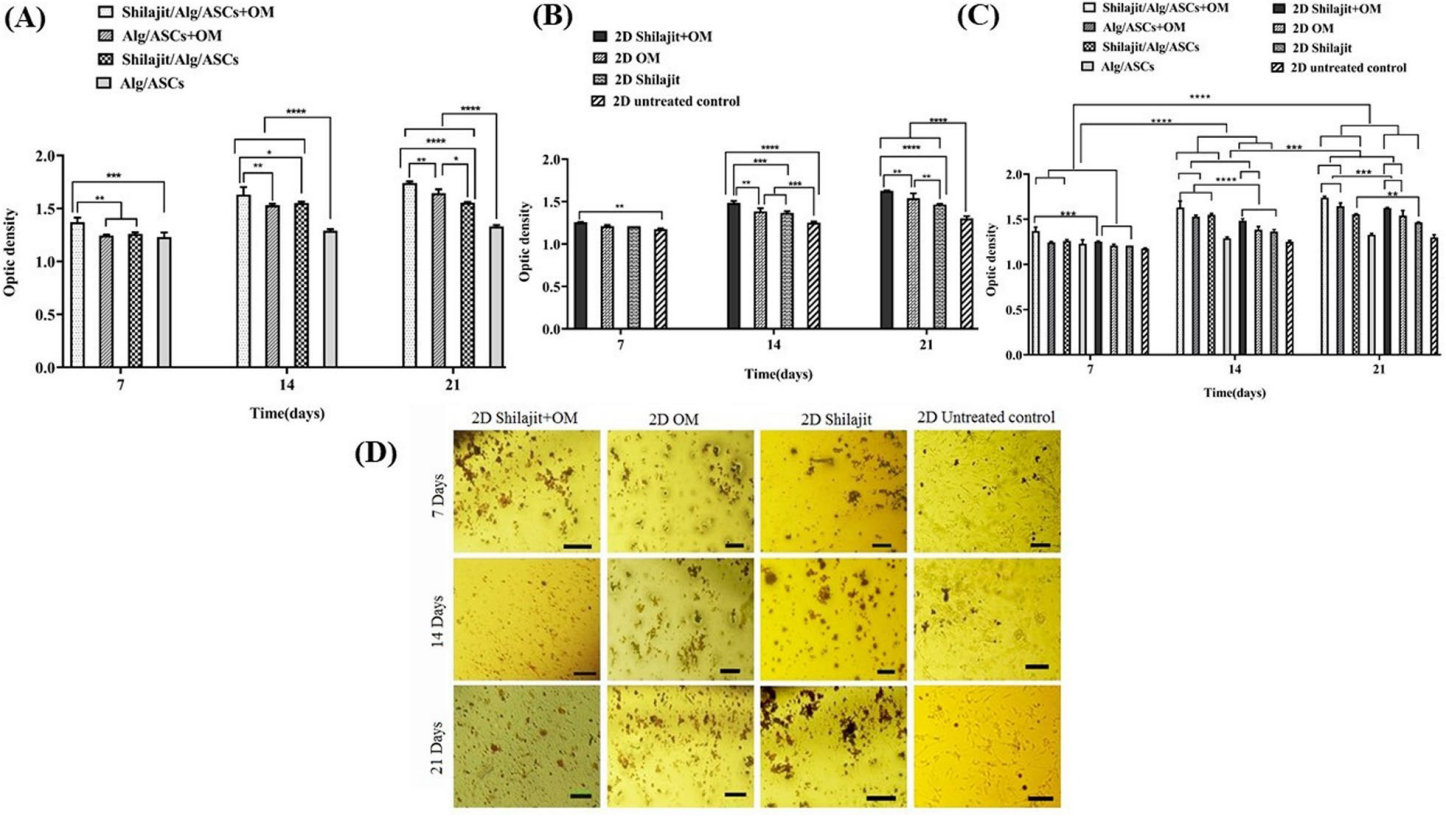
Detection calcium deposition. The calcium deposition assessment was done on 7, 14 and 21 days of differentiation in 3D (**A**) and 2D (**B**) groups and 3D conditions compared with matched 2D cultures in these time points (**C**). The highest level of calcium depositions was seen on Shilajit/Alg/ASCs + OM on 21 days after differentiation compared with other groups. **D** Micrographs of cells stained with alizarin red demonstrate the effects of shilajit on mineral deposition and nodule formation in 2D groups on 7, 14 and 21 days after differentiation. Qualitative observations showed that the number and size of the nodules increased in the presence of Shilajit (*P < 0.05, **P < 0.01, ***P < 0.001, ****P < 0.0001)

Alizarin red S staining was performed to evaluate calcium deposition. The results indicated that the highest calcium was deposited in Shilajit/Alg/ASCs + OM at each time point. In 3D conditions, the accumulation of calcium significantly increased in the Shilajit/Alg/ASCs + OM compared to Alg/ASCs + OM (P = 0.0015), Shilajit/Alg/ASCs (P = 0.003) and Alg/ASCs (P = 0.0006) on day 7. The same trend was also found on days 14 and 21, so that the cells in 3D cultures and in the presence of both Shilajit and OM deposited significantly more calcium compared the cultures deprived either Shilajit (P = 0.0006 and P = 0.008 for 14 and 21 days; respectively) or OM (P = 0.02 and P < 0.0001 for 14 and 21 days, respectively). Besides, on 14 and 21 days, the least calcium deposition belongs to the negative control (P < 0.0001, Fig. 6A).

In the same way with 3D conditions, in the 2D conditions, exposure the cells with a combination of OM and Shilajit resulted a significantly higher level of calcium deposition compared to their counterpart deprived either Shilajit or OM (for 14 days *P* = 0.0012 and *P* = 0.0003, respectively; for 21 days P < 0.01 and P < 0.0001, respectively). Besides, adding either Shilajit or OM alone also boosted the mineralization compared to negative control at day14 (both P < 0.001) and 21(both P < 0.0001). In addition, an obvious increasing in mineralization was done by ASCs cultured in OM compared to the cells exposed Shilajit 21 day (P < 0.01, Fig. 6B).

3D conditions provided a superior environment for calcium deposition, so that calcium content was significantly higher in both Shilajit containing cultures and Alg/ASCs + OM compared to the matched 2D cultures on days 14 (P < 0.0001) and 21 (P = 0.0003, P = 0.003 and P = 0.0008, respectively); however, on day 7, Shilajit could just increase the calcium deposition in 3D cultures at the presence of OM; therefore, the higher calcium content was found in the Shilajit/Alg/ASCs + OM compared to matched 2D cultures treated with Shilajit and OM (P = 0.0003). The calcium deposition also significantly increased in 3D and 2D cultures in all groups as the time progress, so, more calcium was deposited on day 21 compared to the same groups on day 7 (P < 0.0001). Similarly, enhancement of calcium deposition was observed in 3D and 2D cultures in the presence of both Shilajit and OM or only OM on day 21 compared to the same groups on day 14 (P < 0.001 and P < 0.0001, respectively, Fig. 6C).

The microscopic images also confirmed that the calcium deposition was not started massively on day 7 and, the calcium amount was similar in all samples. On day 14 and 21, the most intense staining was observed in the cultures supplemented with OM, Shilajit, or the combination of both with the highest intense staining in the last one. At 21 days, nodule size of calcium displayed a notable increment in all Shilajit compared to the similar cultures on day 14 (Fig. 6D).

### Confocal Raman microscope

**Fig. 7:**
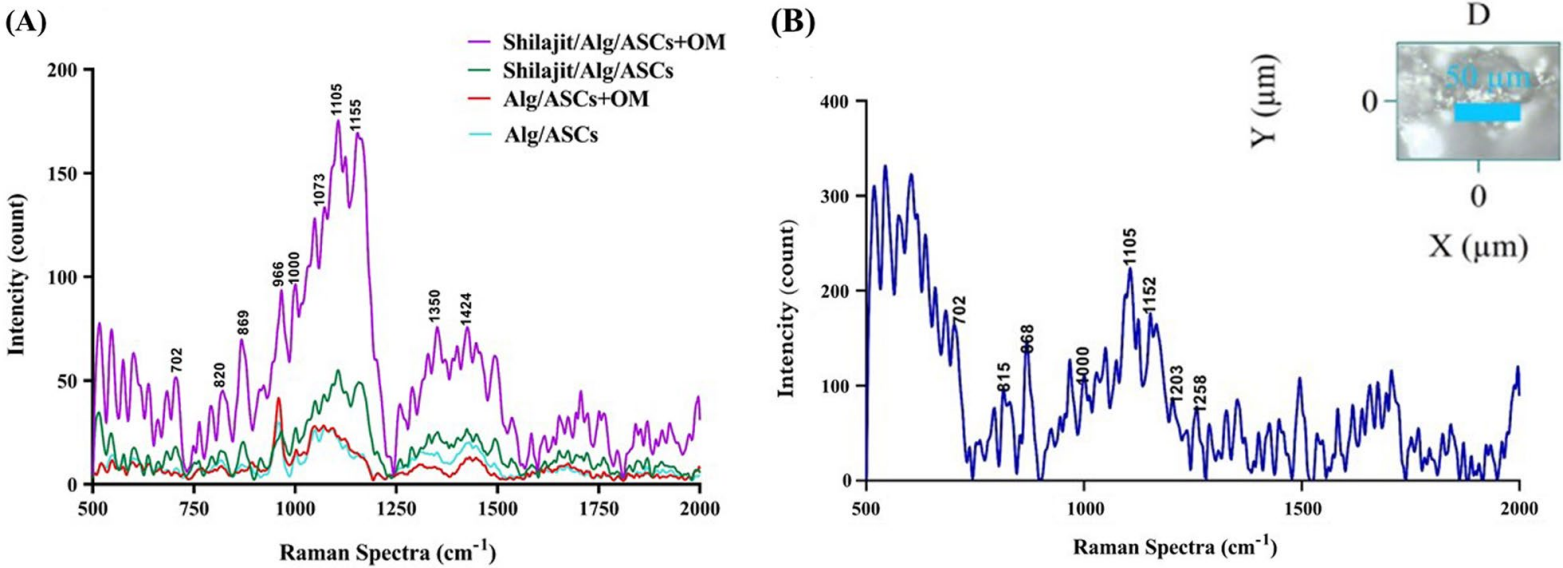
Raman confocal microscopy. **A** Raman spectra from 3D groups 21 days after differentiation show the peaks assigned for mineral and organic compounds related osteogenesis. **B** Raman spectra of Shilajit at 785-nm excitation related to organic and inorganic components at 500–200 cm^−1^

In order to determine the contents of matrix, confocal Raman spectroscopy was randomly executed in four various regions of scaffolds on day 21 after osteogenic induction in 3D groups (Fig. 7A).The Raman peaks corresponding to the different compositions related to the osteogenic differentiation process consisting cholesterol and cholesterol ester at 702 cm^−1^,protein band and proteins such as collagen type I at 820 cm^−1^, hydroxyapatite at 966 cm^−1^, phenylalanine at 1000 cm^−1^,carbonate symmetric stretching vibration of calcium carbonate apatite at 1073 cm^−1^.The peaks for carbohydrate were assigned at 1105 cm^−1^. C–C (&C–N) stretching of proteins (also carotenoids) at1155cm^−1^, carbon particle at 1350 cm^−1^ and deoxyribose at 1424 cm^−1^ were also detected [40]. The results indicated that peak intensity is significantly higher in the Shilajit/Alg/ASCs + OM cultures. The trend of Raman spectra in the Shilajit/Alg/ASCs + OM, Shilajit/Alg/ASC and Alg/ASC + OM was the same.

In addition, confocal Raman spectroscopy was carried out to determine the molecular content of Shilajit (Fig. 7B). A peak at 702 cm^−1^ represents cholesterol and cholesterol ester. Peak at 815 cm^−1^represents proline, hydroxyproline, tyrosine and *v*_2_ PO^−^_2_ stretch of nucleic acid. Peak at 865 cm^−1^is related to C–C stretching, hypro (collagen assignment), monosaccharides (β-fructose), (C–O–C) skeletal mode, disaccharide (sucrose), (C–O–C) skeletal mode polysaccharides, amylose polysaccharides and amylopectin. The Raman spectrum also revealed some peaks corresponding to the different compositions including phenylalanine at 1000 cm^−1^, carbohydrates at 1105 cm^−1^, v(C–N), proteins (protein assignment) v(C–C), carotenoid due to C–C and conjugated C55Cband stretch at 1152 cm^−1^, C–C_6_H_5_ stretch mode (one of C–C ring vibration to be expected in aromatic structure of xylene) at 1203 cm^−1^, amide III, adenine and cytosine at 1258 cm^−1^ [40].

### Scratch wound healing assay (SWH)

**Fig. 8:**
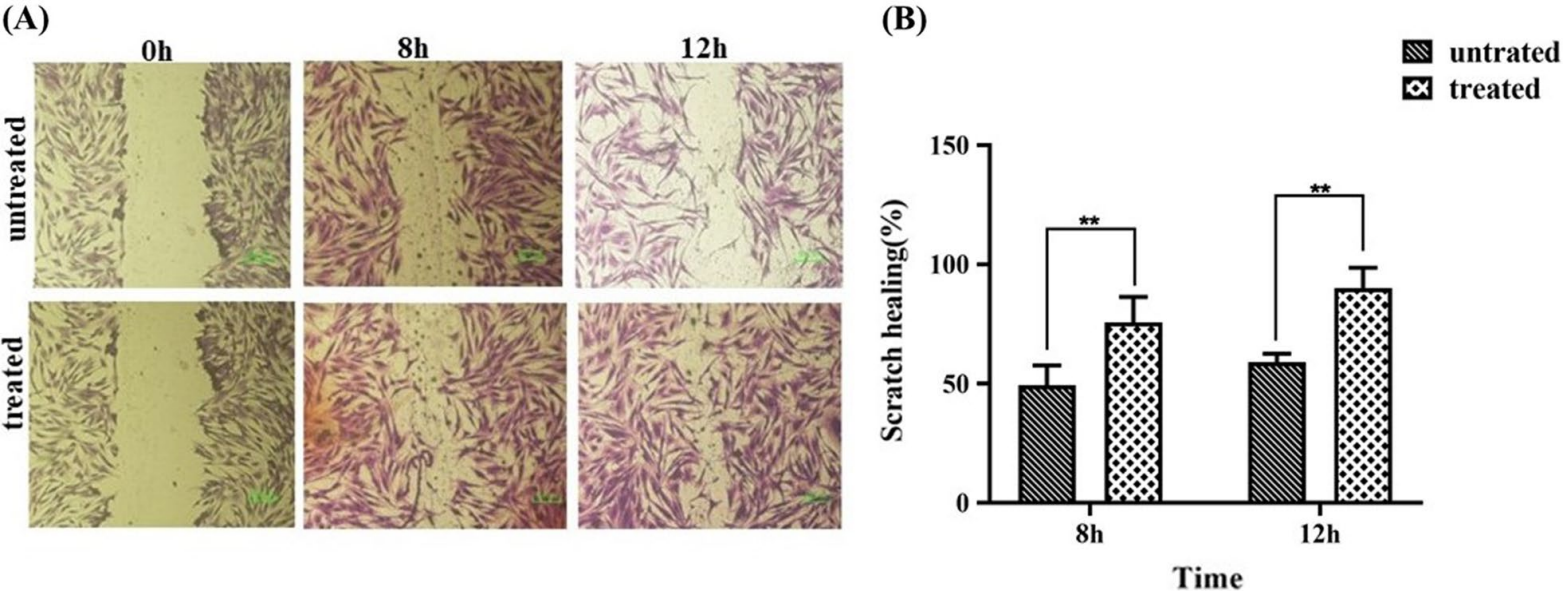
The effect of Shilajit on ASCs proliferation/movement rate in Shilajit treated and untreated groups. **A** Images indicated cell proliferation enhancement at 8 and 12 h post-incubation in both treated and untreated groups compared to the beginning of the test (scale bar: 100 μm). **B** Healing/wounded area ratio in scratch healing analysis showed a significant increase at scratch healing present at treated compared to untreated group at both 8 h and 12 h after treatment. **P < 0.01. **P < 0.01

The effect of Shilajit on ASC proliferation/movement capability was appraised quantitatively by determining healing/wounded area ratio, imitating the wound healing process. Although cell proliferation/movement happens after 8 and 12 h in both treated and untreated groups compared to the beginning of the test (Fig. 8A), a significant elevation of healing was observed in the area of the scratched site of Shilajit treated group compared to untreated group (P = 0.004 and *P* = 0.0016 for 8 and 12 h; respectively) (Fig. 8B).

### Biodegradation

**Fig. 9:**
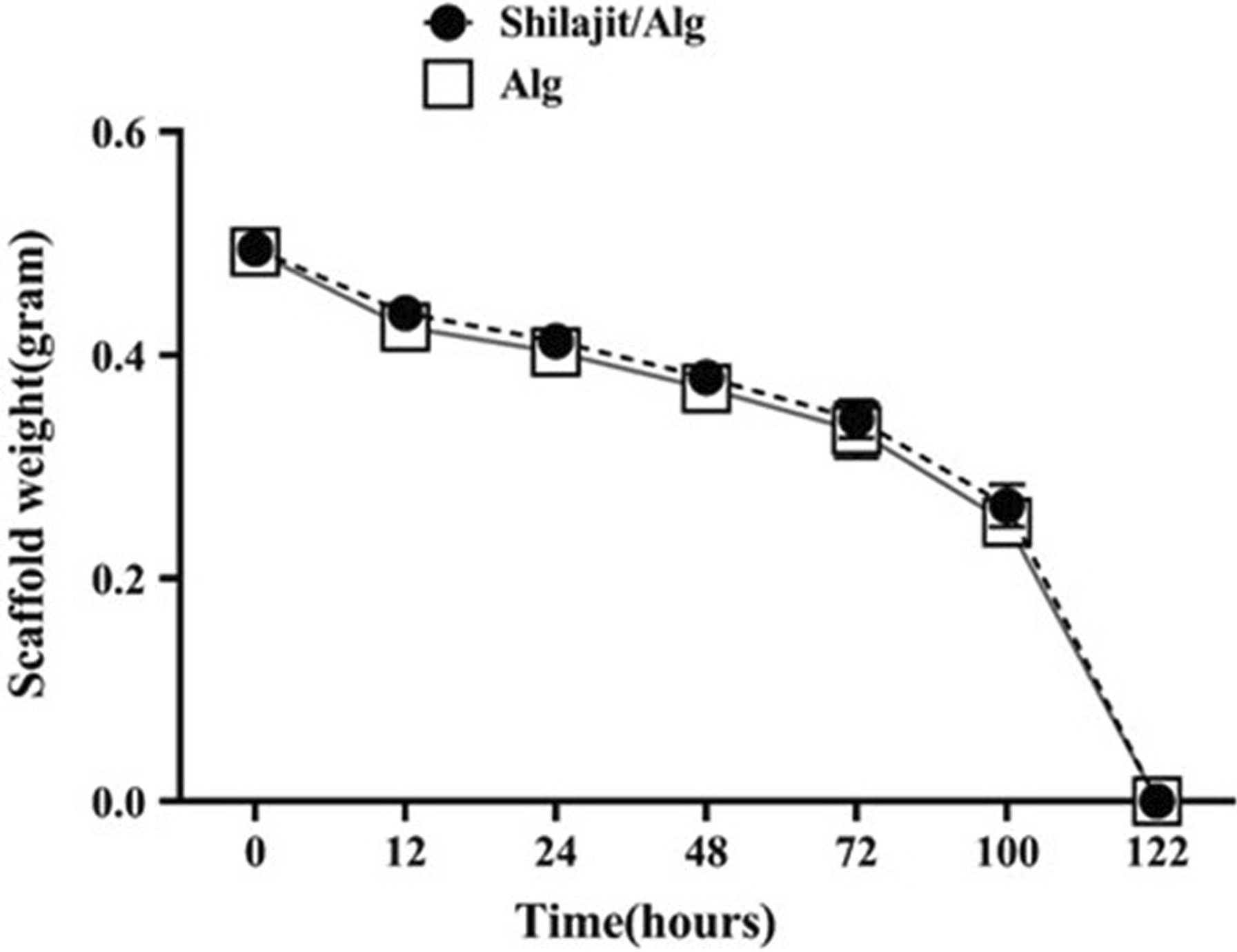
Degradation of scaffolds. Scaffolds prepared with or without Shilajit showed the same degradation rate

The rate of biodegradation of the scaffolds was statistically equal regardless of the presence or absence of Shilajit. After 122 h, both scaffolds were completely degraded. Therefore, Shilajit had no adverse effect on scaffold degradation (Fig. 9).

## Discussion

Exceeding inflammation and calcium content depletion following bone fracture can lead to the elevated chance of damage and lagging bone healing period [29, 41]. One of the main goals of remedy prescription is to achieve bone healing in the shortest time frame using different methods such as therapy with various stem cells without or in combination with a variety of scaffolds such as hydrogels [42]. Among the hydrogels, alginate supports osteogenic differentiation, acts as an exogenous extracellular matrix and is tempting for tissue engineering due to its favorable properties, including biocompatibility, cytocompatibility, biodegradability and ease of gelation [43]. Besides, combining this system with traditional medicine would lead to more outstanding outcomes in regeneration of bone defects.

Here, for the first time, we show that Shilajit in 3D condition promotes osteoblast differentiation of Alg encapsulated ASCs compared to 2D cultures with or without OM. Shilajit not only showed osteoinductive properties but also boosted the osteogenic impact of OM. These findings are in line with what has been indicated for Shilajit in previous studies. Based on in vitro evidence, Shilajit can prevent osteoclastogenesis, while inducing osteoblastic differentiation of MSCs and boost mineralization [39, 44]. Systemic administration of Shilajit to patients with tibial and femoral fracture accelerated bone formation compared to placebo groups [28, 29]. In vivo studies in a rat osteoporotic model revealed that Shilajit may enhance bone formation rate via synthesis of polysaccharides, nucleic acids, and most proteins and hormones needed for cells and tissues growth [45]. Additionally, in line with our findings, in vitro treatment of osteoblast-like cells, MG63 cells, with Shilajit enhanced osteoblast proliferation [45, 46]. Accordingly, based on the in vitro results of our study, Shilajit showed osteoinductive capability as well.

The analysis of Shilajit in the current study represented that it is an appropriate source of elements such as calcium and magnesium. Also, our findings displayed that Shilajit contains biological macromolecules including proteins and amino acids (proline, hydroxyproline, tyrosine, phenylalanine), nucleic acids and nucleotides (adenine and cytosine), carbohydrates (monosaccharides, disaccharides and polysaccharides) and lipids (cholesterol and cholesterol ester). Collagen as one of the most important osteogenic proteins was detected in Shilajit. There are several studies that indicate Shilajit can facilitate bone fracture healing probably because of increasing collagen synthesis, decreasing inflammation and swelling, infection control and improving oxygenation to wound area [18, 47, 48]. Interestingly, ions play prominent roles in regulating many cell functions and biological processes such as cell mitosis, osteogenesis, angiogenesis and antibacterial properties; therefore, they are great applicable in bone regeneration process [49]. Shilajit can be involved in osteogenesis through anabolic activities such as increasing synthesis of proteins and nucleic acids, and transportation of minerals into bone tissue [50]. It has also the potential to induce calcium deposition and promote bone mineralization and osteogenesis [44]. It has been reported that the abundant mineral ingredients, such as calcium and magnesium, play vital roles in the process of osteogenesis. These ions via upregulating the expression of osteopontin, osteocalcin, ALP and collagen type I, inducing osteoblast activity and increasing cell viability can influence bone formation [51–53].

Another interesting approach in regenerative medicine is controlled drug delivery. One of the major mechanisms of release of therapeutic agents in damaged tissue is using degradable materials. Biodegradable property of Alg is an advantageous approach in drug delivery and tissue engineering [54]. Natural and synthetic materials can change the degradation of Alg-based scaffolds. It was displayed that an increase in Aloe Vera content enhances the degradation rate of the alginate hydrogel films [55], whereas adding polyethylene glycol to the alginate-based microcapsules did not affect degradation rate of microcapsules [56]. Based on the results obtained from biodegradation test, Shilajit incorporation had no significant impact on physical property of Alg scaffold. Both Shilajit/Alg combination and Alg had gradually degraded during four days in the same rate and, ultimately both of them completely collapsed after 122 h.

Antioxidant activity is one of the major properties of Shilajit [19], which is confirmed by our data too. Compounds with antioxidant activity as direct scavengers of reactive oxygen species can contribute to the differentiation of osteoblasts and bone formation via inhibition of NF-κB activation and also downregulation of TNF-α and RANKL expression [57]. Therefore, Shilajit can inhibit bone loss and promote bone repair due to its antioxidant activity.

Das et al. (2019) reported that oral consumption of Shilajit can affect skin health and rejuenery by stimulating endothelial cell migration and growth of blood vessels [58]. Likewise, it is showed that Shilajit has rewarding effects on wound healing process in mice model [47]. Our data displayed that Shilajit not only showed any cytotoxic effects on ASCs but also promoted cell proliferation; therefore, we checked the osteogenic induction of the highest dose of Shilajit with the most survival benefit. Also, in the current study, we carried out scratch test to investigate Shilajit effect on ASCs migration. Our data confirmed previous results and showed that Shilajit promotes the healing of scratch area by triggering cell migration and proliferation, whereas treated ASCs maintained spindle-shaped morphology. The migration of endogenous and exogenous MSCs with normal morphology to sites of bone defects is a pivotal issue to start osteogenic differentiation of MSCs and skeletal development. According to researches, the ASCs migration enhancement can induce osteoblast differentiation via upregulation of expression of early growth response gene 1  (EGR-1) and activation of mitogen-activated protein kinases (MAPKs) [59].

## Conclusion

Altogether, Shilajit/Alg scaffold presented a great potential for promoting and accelerating ASCs’ differentiation into osteocyte lineage. Accordingly, this scaffold may provide a new strategy for bone tissue engineering and can be a good therapeutic approach for the treatment of bone defects.

## Data Availability

All data generated or analyzed during this study are included in this published article.

## Abbreviations

ASCs: Human adipose-derived mesenchymal stem cells
2D: Two-dimensional
3D: Three-dimensional
OM: Osteogenic medium
ALP: Alkaline phosphatase
SEM: Scanning electron microscopy
EDX: Energy-dispersive X-ray spectroscopy
Alg: Alginate
MSCs: Mesenchymal stem cells
DBPs: Dibenzo-alpha-pyroons
BM-MSC: Bone marrow-derived mesenchymal stem cells
DMEM: Dulbecco's modified eagle medium
FBS: Fetal bovine serum
PBS: Phosphate-buffered saline
OD: Optical density
